# A comprehensive gene regulatory network for the diauxic shift in *Saccharomyces cerevisiae*

**DOI:** 10.1093/nar/gkt631

**Published:** 2013-07-19

**Authors:** Ludwig Geistlinger, Gergely Csaba, Simon Dirmeier, Robert Küffner, Ralf Zimmer

**Affiliations:** Practical Informatics and Bioinformatics, Institute for Informatics, Ludwig-Maximilians-Universität München, Amalienstrasse 17, 80333 Munich, Germany

## Abstract

Existing machine-readable resources for large-scale gene regulatory networks usually do not provide context information characterizing the activating conditions for a regulation and how targeted genes are affected. Although this information is essentially required for data interpretation, available networks are often restricted to not condition-dependent, non-quantitative, plain binary interactions as derived from high-throughput screens. In this article, we present a comprehensive Petri net based regulatory network that controls the diauxic shift in *Saccharomyces cerevisiae*. For 100 specific enzymatic genes, we collected regulations from public databases as well as identified and manually curated >400 relevant scientific articles. The resulting network consists of >300 multi-input regulatory interactions providing (i) activating conditions for the regulators; (ii) semi-quantitative effects on their targets; and (iii) classification of the experimental evidence. The diauxic shift network compiles widespread distributed regulatory information and is available in an easy-to-use machine-readable form. Additionally, we developed a browsable system organizing the network into pathway maps, which allows to inspect and trace the evidence for each annotated regulation in the model.

## INTRODUCTION

Gene regulatory networks (GRNs) model the effects of transcription factors (TFs) on the expression of their target genes (TGs). As large networks are collected in existing databases, such as RegulonDB (1), YEASTRACT (2) and REDfly (3), it is tempting to use them for the interpretation of large-scale gene and protein expression data.

However, to perform meaningful interpretation of such high-throughput transcriptomic and proteomic data, GRNs need to be modeled at least by (i) defining the conditions under which a regulation takes place or does not take place and (ii) characterizing the effect on the expression of the regulated TG.

The first requirement results from the fact that, to adapt to changing environmental conditions, the cell usually responds with altered gene expression. For example, gene regulation in baker’s yeast *Saccharomyces cerevisiae* changes in response to different nutrients in the growth medium (4). Hence, the interpretion of gene expression measured under certain conditions requires a dynamic condition-dependent definition of the enabled regulations—the active subnetwork of all possible regulations.

The second requirement is due to the fact that genes, qualitatively and quantitatively, are not regulated in a uniform way. On the one hand, again depending on the environmental conditions, relevant genes are activated or repressed to a different extent. On the other hand, combinatorial control of a TG by several TFs can have a non-trivial synergistic effect (5,6). Thus, to understand the observed expression in the data, that is to assign observed expression changes to certain regulators, a detailed characterization of the regulatory effect on the TG expression is necessary. This includes the determination of the ‘effect type’ (activation or inhibition) and the ‘effect strength’ (weak or strong activation/inhibition) as well as an appropriate combination of multi-input effects.

Although both requirements are therefore essential, such context information characterizing a regulation is often unknown or not annotated. Derived from high-throughput protein–protein interaction or TF-binding experiments (7,8), the majority of available large-scale GRNs consists of plain binary interactions, e.g. stating for a certain TF *F* and its TG *G* that *F interacts with G*. The effect of these interactions on gene expression is usually not further characterized. It is also unclear whether the interactions take place under conditions different from the setup used in the respective experiments.

In this article, we propose a model for large-scale GRNs satisfying both requirements and present a comprehensive realization of the model for transcriptional regulation of the diauxic shift in yeast.

*Saccharomyces cerevisiae* is a facultative anaerobic organism preferably fermentating glucose to produce energy for fast growth. Subsequent to the depletion of glucose, fermenting yeasts switch to slower respiratory growth on a non-fermentable carbon source like ethanol, lactate, glycerol or fatty acids. This involves a major reprogramming of gene regulation that includes the deactiviation and activation of specific TFs, which in turn activate or repress specific metabolic genes (1,9,10). Many of the differentially regulated genes code for enzymes, which metabolize the non-fermentable carbon source available in the growth medium, and use the resulting products for the recreation of glucose via gluconeogenesis and the production of energy via the tricarboxylic acid (TCA) cycle.

## MATERIALS AND METHODS

### The yeast GRN

#### Experimental techniques

TFs activate or repress the expression of TGs in response to extra- and intracellular signals. Such gene regulatory interactions (GRIs) between TFs and TGs can be experimentally determined either by directly confirming the TF binding to the regulatory region of the TG or indirectly inferred from TG expression changes following a TF perturbation.

##### Direct evidence (TF binding)

Physical binding of a TF to the promoter of its TG can be determined using several techniques such as wild-type versus TG promoter mutant analysis via a *lacZ*-fusion assay (11) or northern blot (12), DNA footprinting (13), Electrophoretic Mobility Shift Assay (14) and Chromatin ImmunoPrecipitation [ChIP; (15)].

The combination of ChIP with the microarray technology [ChIP-chip; (16)] allows the genome-wide identification of TF-binding sites. ChIP-chip experiments have been comprehensively performed for all yeast TFs (17,18).

**Figure 1.**
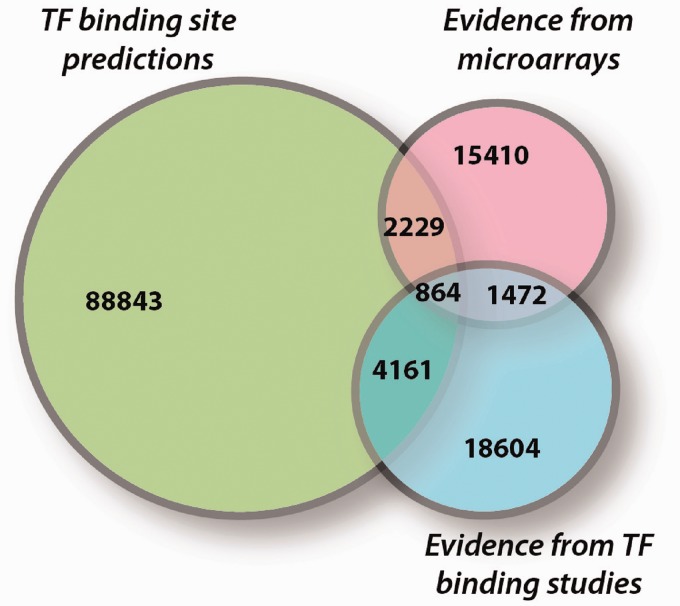
Overlaps between predicted, direct and indirect GRIs in *S. cerevisiae*. GRIs with experimental evidence were taken from YEASTRACT (microarray and binding studies). Predictions were performed for all 160 yeast TF PWMs in JASPAR for the promoter regions of all yeast genes (using the R package *cureos*, default settings). The percentage of predictions, which have an experimental evidence for binding is ∼5.2% (5025 of 96 097). On the other hand, 9.3% (2336 of 25 101) bindings are associated with a change of TG expression.

Putative TGs of a TF can be predicted based on high sequence similarity to its binding sites at the promoter of known TGs. Consensus sequences of TF-binding sites, represented as position weight matrices (PWMs), have been computed for many known yeast TFs and stored in databases like TRANSFAC (19) and JASPAR (20). However, PWM-based GRIs are hypothetical, and only a fraction of them can be experimentally validated (Figure 1).

It is frequently observed that the binding of a certain TF to the promoter of its TG is ineffective, i.e. it does not result in an observable quantitative expression change of the TG. This has several reasons, either other TFs might be required to bind or post-translational modifications (e.g. phosphorylation of the TF) or other signals might be needed to activate the regulatory function of the TF (21). Indeed, as depicted in Figure 1, <10% of known direct physical bindings are associated with a subsequent quantitative fold change of the corresponding TG.

##### Indirect evidence (TG expression)

In contrast to binding studies, regulatory effects (activation or inhibition of TG) can be derived and quantified (fold change) from gene expression studies, where certain TFs have been either knocked out, over-expressed or in other ways functionally modified. Frequently used experimental techniques include *lacZ*-fusion assays, northern blot, real-time PCR (22) and microarrays (23). The most comprehensive series of yeast TF knockout microarrays has been performed by Hu *et al.* (24), where significant expression changes of putative TGs have been assigned to the individual deletion of almost every single yeast TF.

A large fraction of effects observed exclusively in such TF perturbation studies are assumed to be indirect, i.e. the expression change of a TG is a secondary effect, which is due to the deregulation of the TF caused by another knockout. Indeed, <12% of known indirect effects are associated with direct physical binding (Figure 1).

##### Confidence classes of experimental evidence

Whether the confidence in reported GRIs is ‘low’ or ‘high’ depends on the available experimental evidence. Usually, combined evidence of TF binding and TG expression, i.e. the TF binds to the promoter of the TG and a perturbation of the TF results in an expression change of the TG, increases the confidence. In contrast, GRIs with evidence for either binding or expression are not highly reliable *per se* (see again Figure 1). The same holds for additional evidence from consensus analyses and author statements for which the experimental evidence cannot be traced. We thus discriminate in the following between ‘high’ confidence regulations having combined evidence for binding and expression and ‘low’ confidence regulations in all other cases.

#### Resources

**Figure 2.**
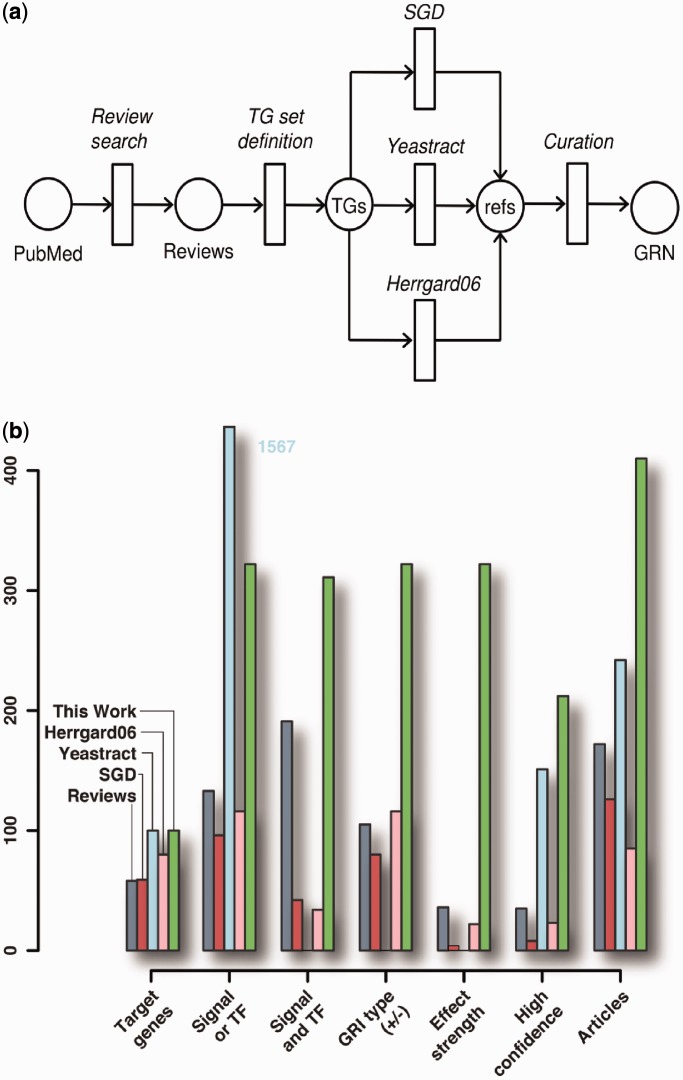
Curation approach. (**a**) Protocol: based on a set of selected reviews, we defined the set of diauxic shift TGs. Each gene was queried for regulatory information in SGD, YEASTRACT and Herrgard *et al.* (26). The GRN was compiled from information directly retrieved from the resources and from the curation of all extracted references. The information collected in each step of our approach is detailed in (**b**). Slots on the *x*-axis from left to right: (1) Number of diauxic shift TGs for which regulatory information could be annotated; (2)–(6) Number of regulations with (2) either Signal *or* TF annotated; (3) Signal *and* TF; (4) regulation type: activation or inhibition; (5) effect strength: weak, medium, strong; (6) High confidence, see ‘Materials and Methods’ section; (7) Number of articles in which regulations could be annotated.

We exploited three representative resources for yeast GRIs: The Sacharomyces Genome Database [SGD; (25)] is the source for a variety of genomic and biological information on *S. cerevisiae* and contains regulatory information for many yeast genes (as quantified in Figure 2b). Besides other widespread biological facts, including post-transcriptional regulation, metabolic function and orthology to genes in other organisms, the SGD summary paragraph on a specific yeast gene often contains different aspects of transcriptional regulation (upstream signals, putative binding sites, validated TF binding, expression effects). However, this valuable information is not easy accessible: the gene summaries are written in free text, and the aspects described differ considerably between genes. Manual curation is thus required to extract this information.

Compared with SGD, YEASTRACT (2) is a specific database for transcriptional regulation in *S. cerevisiae*, in which GRIs are uniformly represented as binary TF–TG associations in a machine-readable format (obtainable as tabular flat file). Mainly derived from recent genome-wide TF binding and TF perturbation experiments, YEASTRACT aims to collect all TFs either binding to a particular TG or show expression changes of the TG when perturbed. Although YEASTRACT stores a large number of GRIs (Figure 1), it does not provide context information characterizing under which conditions the GRIs take place and how targeted genes are affected.

In contrast to YEASTRACT, Herrgard *et al.* (26) have curated the nutrient-controlled regulation of yeast genes involved in metabolic pathways. Mainly derived from detailed studies with a focus on one or a few specific genes, it presumably contains significantly less false-positive GRIs as compared with untargeted genome-wide experiments. Each GRI is classified (activation/inhibition), frequently assigned to a nutrient-based context (extra- and intracellular signals) and described by a boolean rule (e.g. if SIGNAL and TF, then TG). Although enriched with required context information, the GRN is sparse: only a small fraction of the vast amount of articles existing on the regulation of metabolic yeast genes has been taken into account.

### The diauxic shift GRN

#### Curation

We curated a GRN that controls the diauxic shift in three steps (our approach is illustrated in Figure 2a, and the information collected in each step is detailed in Figure 2b):

(1) TG set determination: We collected current reviews on transcriptional regulation of the diauxic shift to define the set of involved TGs. We concentrated on Zaman *et al.* (4), an extensive description of how *Saccharomyces* responds to different nutrients, Hiltunen *et al.* (27) and Gurvitz and Rottensteiner (28) for transcriptional regulation of fatty acid metabolism and oleate induction, and especially on Schüller (9) and Turcotte *et al.* (10), who comprehensively reviewed the transcriptional control of non-fermentative metabolism in *S. cerevisiae*. Based on this literature, we determined the involved metabolic processes and the associated enzymatic TGs.

(2) GRI collection: We systematically queried existing resources, i.e. SGD, YEASTRACT and Herrgard *et al.* (26), for information on the transcriptional regulation of the identified TGs. The representation of the information available in each of the three resources is described in more detail in the previous section.

In SGD, we used the summary site for each TG and manually screened the Description slot and the Summary Paragraph (if existing) for regulatory information. Additionally, we collected all references to the primary literature listed on that page.

In YEASTRACT, we retrieved for each TG all regulating TFs using the Search for TFs (by regulated TG) functionality. From the resulting list of binary TF–TG associations grouped by experimental evidence (direct or indirect, see previous section ‘*Experimental techniques*’), we also collected all references assigned to the associations for experimental support.

Eventually, we restricted the curation of Herrgard *et al.* for all metabolic yeast genes on the information available for the diauxic shift TGs. That yielded a list of regulatory TF–TG relationships (classified as activating or repressing) that are enabled under certain conditions, i.e. triggered by a particular extra- or intracellular signal. Again, we collected all cited references.

(3) GRN compilation: We compiled the GRN via combination of the regulatory information that was directly retrieved from the resources or curated from the references collected in Step 2.

The combination of the directly retrieved information initially required the identification of GRIs contained in two or all three resources. For such well-studied GRIs, the resources often complemented each other. For example, a binary TF–TG association from YEASTRACT could be characterized in more detail with features retrieved from SGD or Herrgard *et al.* such as regulation sign (+/−), effect strength and the enabling context. In addition, GRIs with low confidence in one resource alone frequently gained high confidence when evidence was combined from several resources (see previous section ‘*Confidence classes of experimental evidence*’).

On the other hand, curation of the collected references often allowed the annotation of additional features and a more detailed characterization, especially for poorly studied GRIs contained in only one resource. Curation was performed down to the actual experimental evidence for a GRI under investigation, i.e. references were traced iteratively until the experimental confirmation of the regulation was found. In general, we aimed at the most detailed GRI characterization possible from literature curation, for which we propose a general representation in the next section.

#### Representation

We integrate the curated GRIs into discrete regulation models, i.e. models in which discrete states of the regulators (TFs and signals) result in discrete quantity states of the regulated gene (for instance a low, medium or high expression) depending on the regulation type. We use Petri net models to efficiently represent the information typically available in the literature. Petri nets are well-established graphical and mathematical models (29) and have been extensively applied to biochemical processes, such as signal transduction pathways (30,31) and GRNs (32,33). The extension of Petri net models with fuzzy logic (34) in the PNFL approach (35) allows a more detailed semi-quantitative representation of in- and output of the Petri net transitions, which are defined by simple rule sets according to the regulation type (36,37). Thus, we replace the frequently used representation of GRIs as ‘binary’ TF–TG interactions by ‘multi-input’ Petri net transitions, in which the required context knowledge (activating conditions, combinatorial control and effects on TG expression) can be integrated by accurate parametrization of in- and output and definition of the transition type. The parametrization of such transcriptional transitions is based on a differential regulation setting, where the presence or absence of a signal induces an enhanced or reduced activity of specific TFs, which in turn regulate their TGs differentially (up or down, as compared with the corresponding opposite signal state).

##### Input

The input of a transcriptional transition is composed on the one hand by the context signals, which trigger the regulation and, on the other hand, by the TFs, which perform the actual regulation of the TG under investigation. For example, the depletion of glucose and the availability of a non-fermentable carbon source (the signals) trigger the derepression of enzymes involved in non-fermentative metabolism (the TGs) by specific TFs.

Signals can be extra- or intracellular messenger molecules (such as cAMP), nutritional compositions (for instance, growth media lacking glucose), environmental and experimental conditions (such as high pH or heat stress) and even cellular states (such as retrograde regulation depending on the functional state of the mitochondria).

**Figure 3.**
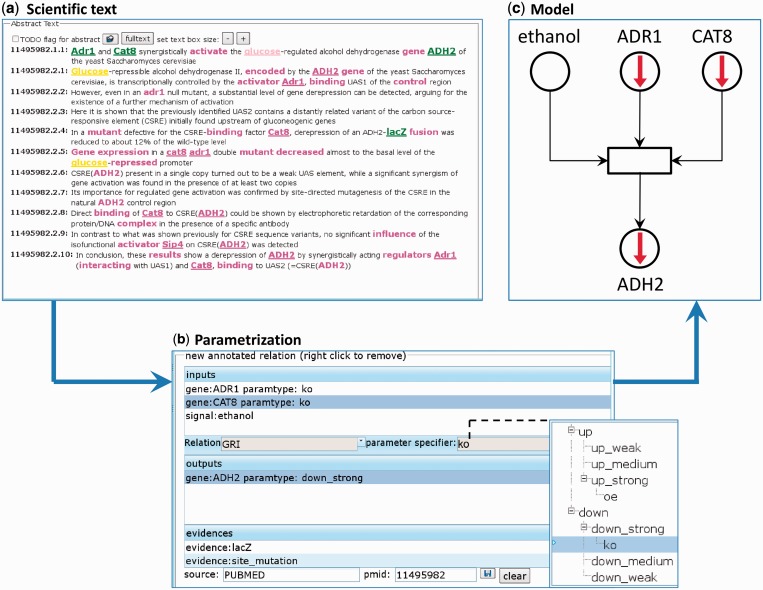
From pure text to semi-quantitative models of GRIs. Within our annotation framework, the pre-indexed regulatory entities in (**a**) can be easily selected and used for the parametrization in (**b**) of input and output of the Petri net model of the GRI in (**c**). In the same manner, experimental evidence can easily assigned to the model.

Based on the absence or presence of given signal(s), the TFs are classified as up- or downregulated in discrete states ‘weak’, ‘medium’ or ‘strong’. The special states ‘overexpression’ (for *up*) and ‘knockout’ (for *down*) were annotated as well (see Figure 3 for an example).

In general, the signal and TF assignments define the conditions under which the transition is enabled.

##### Output

Analogously, TGs are classified as up- or downregulated by a given transition with a ‘weak’, ‘medium’ or ‘strong’ effect strength. Intuitively, this models the fold change in the transcription of the TG. Although the effect strength might differ considerably between data sets in range and distribution, the literature often explicitly states whether a regulation has a weak or strong effect on TG expression. In cases where an exact fold change is reported, we discretize the fold change according to empiric standards: ‘weak’ regulation refers to expression changes below 2-fold, ‘medium’ between 2- and 5-fold and ‘strong’ above 5-fold. The transition type results immediately from a given in- and output configuration, e.g. a TF knockout, resulting in a weak upregulation of the TG, indicates a weak inhibition.

#### Annotation framework

To collect regulatory knowledge effectively from publications, we performed the curation using our in-house annotation software RelAnn (Csaba *et al.*, unpublished). The web-based tool was developed for general text-based annotations of different kinds of relations within a systematic framework.

The main design principles of RelAnn are as follows:

- pre-indexing of defined biological entities (genes, proteins, etc.) in the literature;
- simple click-based annotations to relate the entities to each other; and
- representation of relations as Petri net transitions.

As illustrated in Figure 3, we use RelAnn for the transformation of literature knowledge to the representation of GRIs as semi-quantitative Petri net transitions (as described in the previous ‘Representation’ section).

Subsequent to the pre-indexing of the relevant text using a named entity search, occurrences of defined entities are used for the definition of input (regulators, i.e. TFs and signals), output (regulatees, i.e. TGs) and experimental evidence for a regulatory transition. Thus, every part of the transition (regulatory, regulatees, evidence) is linked to some phrase in a scientific article of the PUBMED database, thereby making the source of the knowledge traceable. In addition, in- and output specification allow the assignment of the semi-quantitative type of needed (input) or induced (output) change associated to the regulation, to wit ‘up’ or ‘down’, with ‘weak’, ‘medium’ or ‘strong’ effect strength (bottom right of Figure 3b).

A special feature of RelAnn is the organization of all components (gene, signal, evidence, regulation and parameter types) in ontologies enabling powerful queries and specifications using generalization and specialization. For example, the regulation annotated in Figure 3 can be not only captured by searching for all regulations having *ethanol* as input, but also by searching for all regulations having a *non-fermentable carbon source* as input.

## RESULTS

### Descriptive analysis

We have curated a gene regulatory network for the diauxic shift in *S. cerevisiae*. As illustrated in Table 1, the curation yielded 1133 text-based annotations of regulatory interactions in 410 scientific articles. The resulting 322 GRIs cover the core processes, taking place during the switch from fermentation to respiration. This includes the regeneration of fermentable glucose (gluconeogenesis), oxidation of glycolytic products (TCA cycle) and catabolism of non-fermentable carbon sources (ethanol, glycerol, lactate, acetate and fatty acids). In addition, we characterized the upstream regulation events of glucose signaling and the corresponding transcriptional regulation of the key signal proteins that act once glucose is depleted. Our network connects 100 TGs with 72 regulating TFs driving the transcriptional response to >50 different extra- and intracellular signal classes. The transcriptional regulation of the regulators themselves has been investigated and integrated into the network.

**Table 1.** Annotation summary

	Genes	TFs	Interactions		Annotations	Articles
			High^a^	All		
Total	100	68	212	322	1133	410
Gluconeogenesis	18	37	56	77	252	117
Fatty acid metabolism	19	20	34	57	203	79
TCA cycle	23	24	29	52	146	64
Glyoxylate cycle	5	27	16	26	102	67
Ethanol metabolism	5	17	13	16	108	76
Glycerol metabolism	3	19	9	18	36	24
Lactate metabolism	3	10	11	11	38	21
Glucose signaling	11	22	25	35	147	71
TF-TF	14	24	19	31	101	69

Shown are the numbers for the total annotation outcome and for the corresponding subprocesses of the diauxic shift. TF: Transcription Factor.

^a^*High*-confidence gene regulatory interactions have experimental evidence for binding and expression (*Materials and Methods*). *All* interactions include *low*- and *high*-confidence interactions.

To estimate the completeness of our network, we extrapolated the expected number of interactions contained in an infinite number of articles relevant for the diauxic shift. Using a first order Hill equation, we estimated that our network is 71% complete (Supplementary Figure S1). The curation of further articles is expected to increase the network size only slightly. For instance, doubling the number of articles by curating 410 additional articles would increase the completeness by just 11 percentage points.

### Visualization

**Figure 4.**
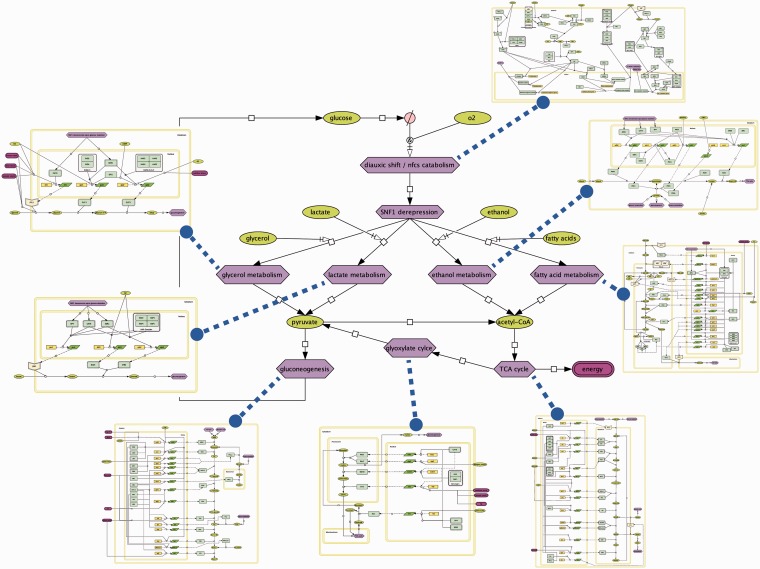
The diauxic shift and its subprocesses. On depletion of glucose, yeast switches from fermentation to respiratory growth on non-fermentable carbon sources such as glycerol, lactate, ethanol and fatty acids. Resulting pyruvate and acetyl-CoA is used to restore glucose and produce energy via gluconeogenesis and the TCA cycle, respectively. As described in the main text, we created pathway maps for each involved subprocess using CellDesigner (38). The maps are organized as exemplarily depicted in Figure 5, and each regulation is clickable and connected to the corresponding annotations designed in our annotation system (Figure 3), enabling a seamless tracing of the evidence from the schematic representation of a regulation in one of the maps down to the exact place in the curated literature.

The systematic Petri net representation of GRIs in our annotation framework is visualized in schematic flowcharts of the subprocesses of the diauxic shift (Figure 4), which we created using the CellDesigner software (38).

**Figure 5.**
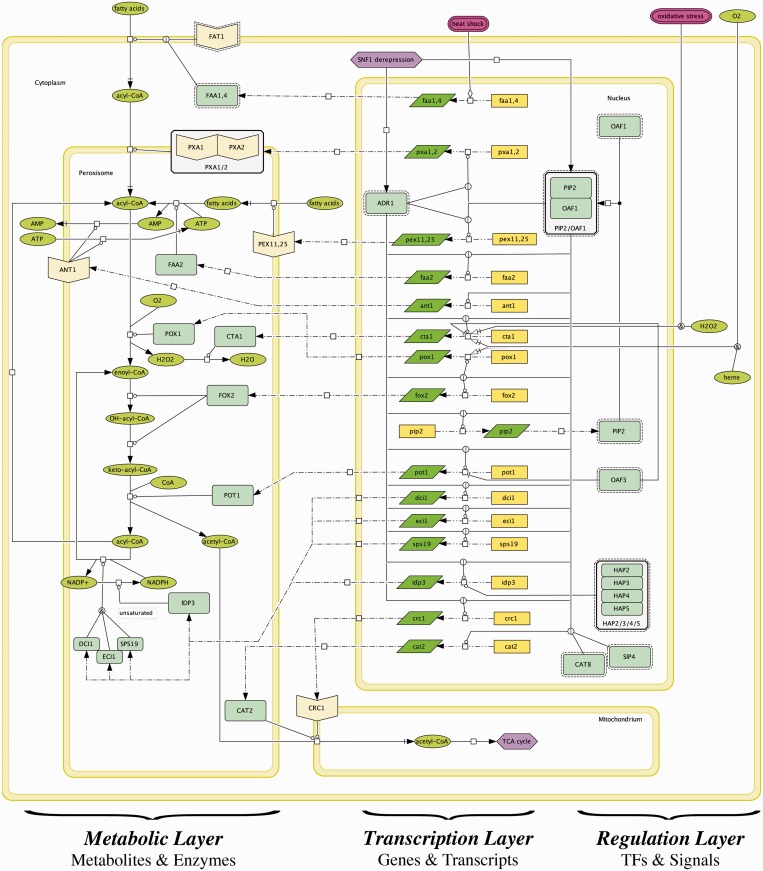
Pathway map of fatty acid metabolism. The map is compartmentalized (cytoplasm, peroxisome, mitochondrium and nucleus) and composed from three layers: the regulation layer on the right, which contains the TFs (light green rectangles) and the signals (green and purple ellipses for metabolites and conditions, respectively) that govern the transcription of genes (yellow rectangles) to their corresponding transcripts (green rhomboids) in the middle. The metabolic layer on the left depicts the translated enzymes (light green rectangles) that catalyze the interconversion of substrates and products (green ellipses), some of which are needed or produced from other subprocesses (blue hexagons) of the diauxic shift.

As exemplarily illustrated for the metabolism of fatty acids in Figure 5, the pathway maps are structured by a regulation, transcription and metabolic layer assigned to different cell compartments (cytoplasm, nucleus, peroxisome and mitochondrium). Thus, the maps are not restricted to the pure illustration of the signals and TFs (regulation layer) regulating the transcription of the mostly enzymatic genes to the corresponding mRNA transcripts (transcription layer), but they also visualize the metabolic reactions that are subject to the transcriptional control.

Each transcriptional transition in the CellDesigner maps is clickable and connected to the corresponding annotations, enabling a seamless tracing of the evidence from the schematic representation of a regulation in one of the maps down to the exact place in the curated literature. The interactive network can be accessed under http://services.bio.ifi.lmu.de/diauxicGRN.

### Comparison to existing resources

#### General comparison

Existing resources on the transcriptional regulation in *S. cerevisiae* differ considerably in the way how GRIs are represented (see ‘Materials and Methods’ section for an overview). Concentrating on the diauxic shift, we combined and extended the representations in SGD (25), YEASTRACT (2) and Herrgard *et al.* (26), especially improving on three major aspects:

1. *Context*, determination of the conditions under which a regulation is enabled
2. *Effect*, characterization of regulation type and strength
3. *Evidence*, collection and classification of experimental support

Considering these aspects, the amount of information that the respective resources provided in each step of our curation approach is illustrated in Figure 2.

Of the 100 genes classified beforehand as relevant for the diauxic shift (see ‘Materials and Methods’ section), SGD provides regulatory information on 59 genes, Herrgard *et al.* on 80 genes and YEASTRACT on all 100 genes. A resource is defined to provide regulatory information on a gene, if it either has a regulating signal *or* TF assigned.

In YEASTRACT, each diauxic shift gene has a number of regulating TFs annotated, yielding in total 1567 binary interactions (i.e. one-to-one TF:TG associations). Context information, such as extra- or intracellular signals, which turn the regulating TFs active, is not available. However, this is an essential aspect as the transcriptional response of yeast to different environmental conditions varies drastically (39), and most yeast TFs are known to change their activity in dependence on the environmental conditions (18). In SGD and Herrgard *et al.* (26), the fraction of regulations with thorough context definition (signal *and* TF) out of all regulations with signal *or* TF is small (44 and 29%, respectively). In contrast, the regulations in our network have a context annotation in >96% of the cases.

Second, we characterized the regulatory effect in more detail via annotation of the effect type and strength. That means we determined whether a regulation results in a weak, medium or strong activation or inhibition of the affected gene. This feature enables a more fine-grained interpretation and prediction of the expression change of a target in dependence on the TF activity. Herrgard *et al.* and SGD typically provide regulations with an annotated effect type (activation/inhibition), whereas YEASTRACT does not distinguish between different interaction types. The semi-quantitative characterization of the effect strength is a novel feature of our network, and little is annotated here in other resources.

Third, we designed a classification to judge how reliable the experimental evidence of a regulatory interaction is. As defined in ‘Materials and Methods’ section, a regulation with ‘high’ confidence is given if the corresponding TF has been experimentally determined to bind to the promoter of its target and the target is expressed differentially when the TF is perturbed. Our network contains 66% interactions with high confidence, compared with <10% in the other resources.

Concentrating on the diauxic shift genes, our work is based on by far the largest number of articles in which regulations of these genes could be annotated (410 articles, compared with 242, 126 and 85 articles by YEASTRACT, SGD and Herrgard *et al.*, respectively). Although this implies that the quantity of curated articles is crucial for a comprehensive characterization, it is also important which articles are considered. Interestingly, we observed that the five review articles on transcriptional regulation of the diauxic shift (see ‘Materials and Methods’ section) provide more regulatory information than SGD (see again Figure 2).

#### PCK1 example

**Figure 6.**
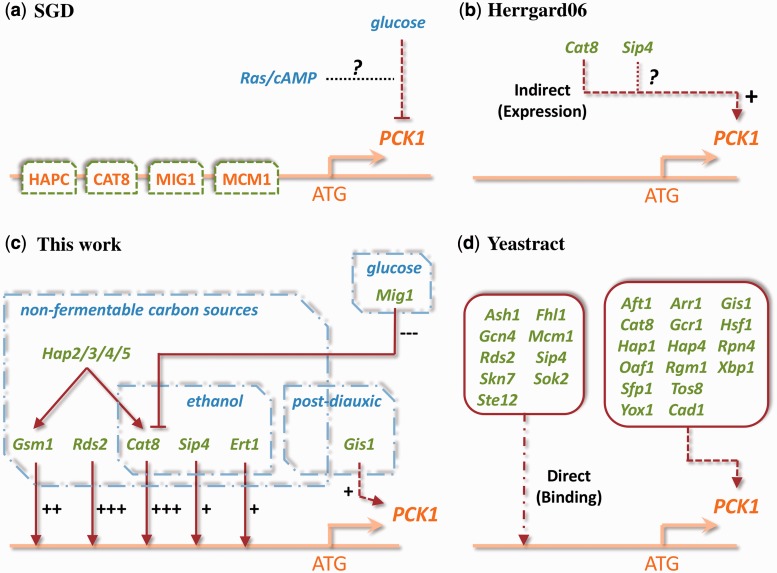
Current representations of PCK1 regulation. (**a**) SGD states that the PCK1 upstream region contains consensus binding sites for MIG1, the HAP complex, CAT8 and MCM1. Further, that PCK1 is glucose-repressed, which seems to be mediated by Ras/cAMP; (**b**) Herrgard *et al.* state that PCK1 is CAT8/SIP4 activated, with expression evidence for the activation by CAT8; however, no annotated evidence for the activation by SIP4; (**c**) Our work extends current views by accurate context assignment and effect characterization (+/−) and quantification (weak, medium, strong) for each regulation; (**d**) YEASTRACT lists a variety of direct and indirect effects, which are not further detailed. In addition, combined evidence is collected for strong confidence regulations (binding and expression; continuous lines) and weak confidence regulations (binding or expression only; dashed lines).

Considering the example of PCK1 regulation, a key enzyme of gluconeogenesis, the differences in the three existing resources—with respect of the three aspects *context*, *effect* and *evidence* elucidated in the previous section—are illustrated in Figure 6.

The SGD notes, besides a variety of biological information on PCK1, putative binding sites for the TFs MIG1, CAT8, MCM1 and the HAP complex. Furthermore, it is stated that glucose represses PCK1 expression, which seems to be mediated by Ras/cAMP signaling.

YEASTRACT yields a relatively large number of additional TFs experimentally determined to bind to the PCK1 promoter, and TFs for which PCK1 shows a differential expression in TF mutant versus wild-type analyses. Herrgard *et al.* lists that CAT8 and SIP4 activate PCK1. As explained earlier in the text, we extended the current representations of PCK1 regulation as follows.

First, we performed an accurate context assignment. In the PCK1 example, SGD, YEASTRACT and Herrgard *et al.* indicate that CAT8 regulates PCK1. However, this regulation takes place only during growth on non-fermentable carbon sources, in particular on ethanol (10)—a crucial context information only included in our work (Figure 6c). As CAT8 is inactive under standard conditions (glucose medium), a CAT8 knockout would not influence PCK1 expression at all (21).

Second, we discriminate for all regulations in our network between weak, medium or strong activation and inhibition (correspondingly depicted as +/++/+++ and −/−−/−−− in Figure 6c).

Third, we classified the experimental evidence for a regulation to have low or high confidence to distinguish biological regulation *in vivo* from ineffective or indirect regulation. In the PCK1 example, all regulatory interactions from SGD, YEASTRACT and Herrgard *et al.* have low confidence *per se*. The four putative TF-binding sites in the PCK1 promoter mentioned by SGD are not experimentally confirmed by a binding technique like ChIP (see ‘Materials and Methods’ section). In YEASTRACT, one subset of TFs is shown to bind PCK1, but a regulatory effect on expression of PCK1 is not annotated. Vice versa, the subset of TFs annotated to have an expression effect lacks information on binding. Similarly, Herrgard *et al.* (26) cites an expression study for the regulation of PCK1 by CAT8; for regulation by SIP4 no evidence is annotated.

In part, we increased the confidence in these regulations by collecting additional evidence (for CAT8, SIP4 and RDS2). We also identified new regulations with high confidence (ERT1 and GSM1) by directly querying PUBMED for regulation of PCK1. On the other hand, we determined regulations that are indirect. For example, the HAP2/3/4/5 activator complex and the MIG1 glucose repressor act indirectly via regulation of CAT8, rather than by direct regulation of PCK1 (10). Lastly, we discarded putative regulators not biologically plausible to regulate PCK1, i.e. TFs known to exclusively regulate targets functionally unrelated to PCK1. These are presumably false positives from high-throughput experiments (e.g. ASH1, GCN4 and STE12).

## DISCUSSION

The gene regulatory network of baker’s yeast *S. cerevisiae* has been comprehensively studied during the past decades. To provide a machine-readable review of the current diauxic shift knowledge and to investigate how it could be represented to model the regulation of important molecular processes, we addressed the following questions:

1. Do the existing resources already fully characterize the regulation of a given process?
2. If not, how can such a comprehensive characterization be achieved?
3. Which level of granularity is best suited to represent the volume and detail of the available heterogeneous information?

For these questions, we considered different representative resources such as the SGD (25). SGD provides, for one gene at a time, a brief summary of major regulatory impacts such as extra- and intracellular signals. YEASTRACT (2), on the other hand, is a repository for binary GRIs (i.e. one-to-one TF:TG associations), mainly derived from published high-throughput TF binding (18) and perturbation experiments (24). In contrast, Herrgard *et al.* (26) have manually curated the transcriptional regulation of metabolic yeast genes in more detail from the literature, annotating additional features such as the interaction type (activation or inhibition).

As all three resources have a different focus, they thus provide characteristic information on different aspects of gene regulation that we combined to obtain a more complete picture. Thus, we first evaluated to which extent the integration of the heterogeneous resources yields a comprehensive yet detailed characterization of a process-scale gene regulatory network. As a showcase, we chose the particularly well-studied transcriptional regulation of switching from fermentation to respiration, the diauxic shift in yeast.

Based on current reviews on transcriptional regulation of the diauxic shift, we defined the set of ∼100 TGs whose gene products perform relevant steps of the shift such as the enzymatic conversion of metabolites. For this gene set, we aimed to retrieve details on their regulation from the three resources. That involves not only the regulators affecting a given TG but also the conditions under which the TG is affected and whether the gene is activated or inhibited by this relationship. Although a large number of raw binary TF:TG regulatory interactions can be obtained from YEASTRACT, their corresponding context information necessary for a detailed understanding of the interaction could only partially annotated using information from SGD and Herrgard *et al.* Although each of the three resources cited a large part of the relevant literature as evidence for the regulations, they did not fully exploit the regulatory context information described in the literature. Consequently, thorough manual re-curation of the cited scientific articles, i.e. the full text including tables and figures, was necessary to obtain the activation context of the regulator(s), potential interplay between regulators, the regulation type (activation or inhibition) and the experimental evidence.

We thus dealt with the first two questions by performing a hierarchical curation approach whereby we compiled a comprehensive set of process-relevant genes, extracted and integrated the regulatory information available for these genes from current databases and resources, and finally complemented the obtained regulatory interactions by a thorough manual literature curation.

We estimate that our network, result of an exhaustive databases and literature search, captures >70% of the complete regulatory network affecting genes involved in the diauxic shift. Covering each interaction more than three times on average, we reached a saturation degree that it would, by extrapolation, need twice the number of currently considered articles to achieve 80% completeness.

Efficiently scaling up from process-specific to organism-wide regulatory networks requires authors and data resources to accurately and uniformly annotate context information when reporting gene regulatory information. Using established machine-readable formats like SBML (40) would then allow a semi-automated processing in which expert intervention and curation is only necessary when compiling regulatory information from conflicting studies.

Addressing the third question, we compiled information on the activation context of TFs and their effect strength on their targets. The latter is often stated in terms of fold changes or discrete quantity changes of the TGs (e.g. ‘In a yeast strain deleted for ADR1, expression of ADH2 was found to be *strongly* decreased.’). Although such semi-quantitative information was abundantly found in the literature, kinetic parameters as required for quantitative modeling with ordinary differential equations (ODEs) were only rarely reported.

We therefore suggested an intermediate representation of GRIs that is beyond current coarse-grained purely qualitative characterization; on the other hand, of course, it does not match the fine-grained quantitative ODE models.

In such a representation, an interaction between one or more TFs and a TG is characterized in dependence on the activation context of the TFs and by the semi-quantitative effect on corresponding TGs. This seems to strike the balance between striving for a detailed model granularity, and optimally and comprehensively exploiting the available knowledge on the other hand. This also enables a model-based data view, i.e. the model can be tested whether the annotated, and thus expected, behavior of regulations agrees with the observed behavior in a particular data set of gene expression measurements under investigation.

The suggested representation is exploited in our resulting diauxic shift network, comprising >300 multi-input regulations that also account for combinatorial control by more than one regulator. Available in a machine-readable flat format, it is readily usable in network-based approaches for the interpretation of gene expression data. As a front end, we further provide interactive pathways maps, enabling intuitive exploration of the network modules integrated into our annotation system, where the evidence for each regulation can be entered or retrieved down to the exact reference position in the primary literature. Our system can serve as a starting point to similarly annotate and incorporate additional processes, e.g. all processes subject to glucose control, as the addition of new annotations to existing transitions and pathway maps is straightforward and can be interconnected to the already existing maps.

The system and all accompanying resources are available under http://services.bio.ifi.lmu.de/diauxicGRN.

## SUPPLEMENTARY DATA

Supplementary Data are available at NAR Online.

## FUNDING

DFG international research training group [1563/1 RECESS to L.G.]. Funding for open access charge: Ludwig-Maximilians-Universität München and RECESS.

*Conflict of interest statement.* None declared.

## Supplementary Material

Supplementary Data

## References

[1] Gama-Castro S, Salgado H, Peralta-Gil M, Santos-Zavaleta A, Muniz-Rascado L, Solano-Lira H, Jimenez-Jacinto V, Weiss V, Garcia-Sotelo JS, Lopez-Fuentes A (2011). RegulonDB version 7.0: transcriptional regulation of *Escherichia coli* K-12 integrated within genetic sensory response units (Gensor Units). Nucleic Acids Res..

[2] Abdulrehman D, Monteiro PT, Teixeira MC, Mira NP, Lourenco AB, dos Santos SC, Cabrito TR, Francisco AP, Madeira SC, Aires RS (2011). YEASTRACT: providing a programmatic access to curated transcriptional regulatory associations in *Saccharomyces cerevisiae* through a web services interface. Nucleic Acids Res..

[3] Gallo SM, Gerrard DT, Miner D, Simich M, Des Soye B, Bergman CM, Halfon MS (2011). REDfly v3.0: toward a comprehensive database of transcriptional regulatory elements in *Drosophila*. Nucleic Acids Res..

[4] Zaman S, Lippman SI, Zhao X, Broach JR (2008). How *Saccharomyces* responds to nutrients. Annu. Rev. Genet..

[5] Kel OV, Romaschenko AG, Kel AE, Wingender E, Kolchanov NA (1995). A compilation of composite regulatory elements affecting gene transcription in vertebrates. Nucleic Acids Res..

[6] Balaji S, Babu MM, Iyer LM, Luscombe NM, Aravind L (2006). Comprehensive analysis of combinatorial regulation using the transcriptional regulatory network of yeast. J. Mol. Biol..

[7] Walhout AJ (2006). Unraveling transcription regulatory networks by protein-DNA and protein-protein interaction mapping. Genome Res..

[8] Kim TM, Park PJ (2011). Advances in analysis of transcriptional regulatory networks. Wiley Interdiscip Rev. Syst. Biol. Med..

[9] Schüller HJ (2003). Transcriptional control of nonfermentative metabolism in the yeast Saccharomyces cerevisiae. Curr. Genet..

[10] Turcotte B, Liang XB, Robert F, Soontorngun N (2010). Transcriptional regulation of nonfermentable carbon utilization in budding yeast. FEMS Yeast Res..

[11] Miller JH (1972). Experiments in Molecular Genetics.

[12] Alwine JC, Kemp DJ, Stark GR (1977). Method for detection of specific RNAs in agarose gels by transfer to diazobenzyloxymethyl-paper and hybridization with DNA probes. Proc. Natl Acad. Sci. USA.

[13] Galas D, Schmitz A (1978). DNAse footprinting: a simple method for the detection of protein-DNA binding specificity. Nucleic Acids Res..

[14] Garner MM, Revzin A (1981). A gel electrophoresis method for quantifying the binding of proteins to specific DNA regions: application to components of the Escherichia coli lactose operon regulatory system. Nucleic Acids Res..

[15] Collas P (2010). The current state of chromatin immunoprecipitation. Mol. Biotechnol..

[16] Buck MJ, Lieb JD (2004). ChIP-chip: considerations for the design, analysis, and application of genome-wide chromatin immunoprecipitation experiments. Genomics.

[17] Lee TI, Rinaldi NJ, Robert F, Odom DT, Bar-Joseph Z, Gerber GK, Hannett NM, Harbison CT, Thompson CM, Simon I (2002). Transcriptional regulatory networks in *Saccharomyces cerevisiae*. Science.

[18] Harbison CT, Gordon DB, Lee TI, Rinaldi NJ, Macisaac KD, Danford TW, Hannett NM, Tagne JB, Reynolds DB, Yoo J (2004). Transcriptional regulatory code of a eukaryotic genome. Nature.

[19] Wingender E (2008). The TRANSFAC project as an example of framework technology that supports the analysis of genomic regulation. Brief Bioinform..

[20] Portales-Casamar E, Thongjuea S, Kwon AT, Arenillas D, Zhao X, Valen E, Yusuf D, Lenhard B, Wasserman WW, Sandelin A (2010). JASPAR 2010: the greatly expanded open-access database of transcription factor binding profiles. Nucleic Acids Res..

[21] Chua G, Morris QD, Sopko R, Robinson MD, Ryan O, Chan ET, Frey BJ, Andrews BJ, Boone C, Hughes TR (2006). Identifying transcription factor functions and targets by phenotypic activation. Proc. Natl Acad. Sci. USA.

[22] VanGuilder HD, Vrana KE, Freeman WM (2008). Twenty-five years of quantitative PCR for gene expression analysis. Biotechniques.

[23] DeRisi JL, Iyer VR, Brown PO (1997). Exploring the metabolic and genetic control of gene expression on a genomic scale. Science.

[24] Hu Z, Killion PJ, Iyer VR (2007). Genetic reconstruction of a functional transcriptional regulatory network. Nat. Genet..

[25] Skrzypek MS, Hirschman J (2011). Using the *Saccharomyces* genome database (SGD) for analysis of genomic information. Curr. Protoc. Bioinformatics.

[26] Herrgard MJ, Lee BS, Portnoy V, Palsson B (2006). Integrated analysis of regulatory and metabolic networks reveals novel regulatory mechanisms in *Saccharomyces cerevisiae*. Genome Res..

[27] Hiltunen JK, Mursula AM, Rottensteiner H, Wierenga RK, Kastaniotis AJ, Gurvitz A (2003). The biochemistry of peroxisomal beta-oxidation in the yeast *Saccharomyces cerevisiae*. FEMS Microbiol. Rev..

[28] Gurvitz A, Rottensteiner H (2006). The biochemistry of oleate induction: transcriptional upregulation and peroxisome proliferation. Biochim. Biophys. Acta.

[29] Murata T (1989). Petri nets: properties, analysis and applications. Proc. of the IEEE.

[30] Lee DY, Zimmer R, Lee SY, Hanisch D, Park S (2004). Knowledge representation model for systems-level analysis of signal transduction networks. Genome Inform..

[31] Lee DY, Zimmer R, Lee SY, Park S (2005). Colored Petri net modeling and simulation of signal transduction pathways. Metab. Eng..

[32] Koch I, Das S, Caragea D, Welch SM, Hsu WH (2010). Chapter 25: Petri Nets and GRN Models. Handbook of Research on Computational Methodologies in Gene Regulatory Networks.

[33] Koch I, Reisig W, Schreiber F (2010). Modeling in Systems Biology: The Petri net approach.

[34] Zadeh LA (1963). Fuzzy sets. Inform. Control.

[35] Windhager L, Erhard F, Zimmer R, Koch I, Reisig W, Schreiber F (2010). Fuzzy modeling. Modeling in Systems Biology: The Petri net approach.

[36] Küffner R, Petri T, Windhager L, Zimmer R (2010). Petri nets with fuzzy logic (PNFL): reverse engineering and parametrization. PLoS One.

[37] Geistlinger L, Csaba G, Küffner R, Mulder N, Zimmer R (2011). From sets to graphs: towards a realistic enrichment analysis of transcriptomic systems. Bioinformatics.

[38] Funahashi A, Tanimura N, Morohashi M, Kitano H (2003). CellDesigner: a process diagram editor for gene-regulatory and biochemical networks. Biosilico.

[39] Gasch AP, Spellman PT, Kao CM, Carmel-Harel O, Eisen MB, Storz G, Botstein D, Brown PO (2000). Genomic expression programs in the response of yeast cells to environmental changes. Mol. Biol. Cell.

[40] Hucka M, Finney A, Sauro HM, Bolouri H, Doyle JC, Kitano H, Arkin AP, Bornstein BJ, Bray D, Cornish-Bowden A (2003). The systems biology markup language (SBML): a medium for representation and exchange of biochemical network models. Bioinformatics.

