# Supplementary material for: A comprehensive gene regulatory network for the diauxic shift in Saccharomyces cerevisiae
Source: Nucleic Acids Res. 2013 Jul 19;41(18):8452–63. doi: 10.1093/nar/gkt631 (PMC3794591; doi:10.1093/nar/gkt631)
Supplement: Supplementary Data [file supp_41_18_8452__index.html]

A comprehensive gene regulatory network for the diauxic shift in Saccharomyces cerevisiae — A comprehensive gene regulatory network for the diauxic shift in Saccharomyces cerevisiae — Supplementary Data 

# A comprehensive gene regulatory network for the diauxic shift in *Saccharomyces cerevisiae*

## 

files

**Files in this Data Supplement:**

- Supplementary Data - pdf file
