# Supplementary material for: A comprehensive gene regulatory network for the diauxic shift in Saccharomyces cerevisiae
Source: Nucleic Acids Res. 2013 Jul 19;41(18):8452–63. doi: 10.1093/nar/gkt631 (PMC3794591; doi:10.1093/nar/gkt631)
Supplement: Supplementary Data [file supp_gkt631_nar-00921-n-2013-File005.pdf]

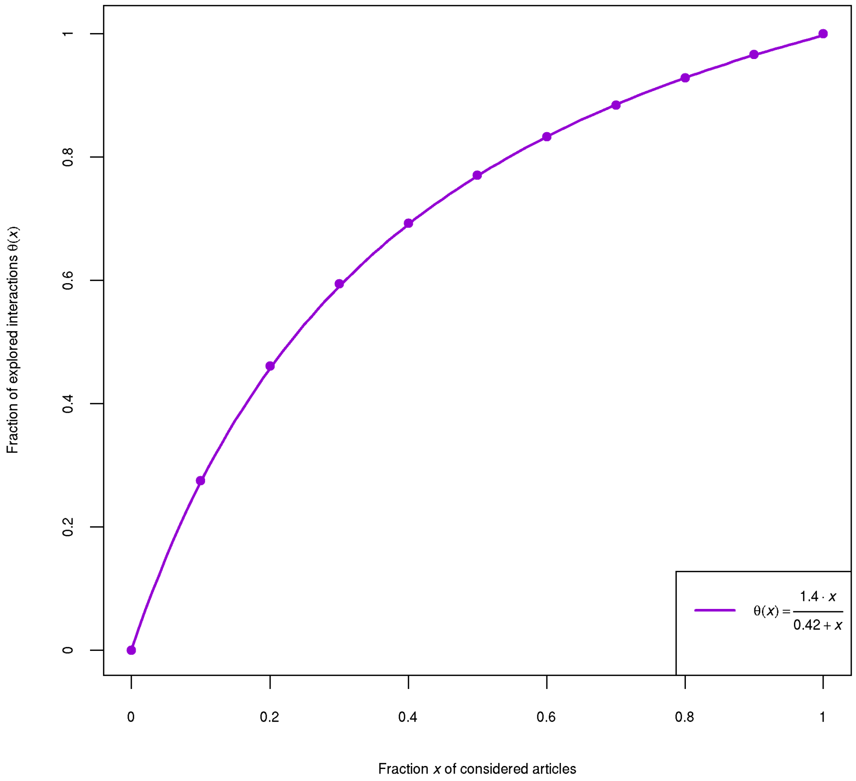

Figure S1: Estimation of the network completeness. Using a first order Hill equation, we estimated that our network is 71% complete (corresponds to a fraction of explored interactions $\theta(x) = 1$). 50% completeness can be achieved with 0.42 times the total number of currently considered articles ($0.42 \cdot 410 = 172$ articles). Based on this estimation, we extrapolated that the curation of 410 additional articles would increase the completeness by 11 percentage points.
